# Delayed macrophage targeting by clodronate liposomes worsens the progression of cytokine storm syndrome

**DOI:** 10.3389/fimmu.2024.1477449

**Published:** 2024-10-28

**Authors:** Kunjan Khanna, Emily Eul, Hui Yan, Roberta Faccio

**Affiliations:** ^1^ Department of Orthopedics, Washington University in St Louis, St Louis, MO, United States; ^2^ Shriners Hospital for Children, St Louis, MO, United States

**Keywords:** clodronate, cytokine storm syndrome (CSS), inflammation, macrophages, TIM4

## Abstract

Excessive macrophage activation and production of pro-inflammatory cytokines are hallmarks of the Cytokine Storm Syndrome (CSS), a lethal condition triggered by sepsis, autoimmune disorders, and cancer immunotherapies. While depletion of macrophages at disease onset protects from lethality in an infection-induced CSS murine model, patients are rarely diagnosed early, hence the need to characterize macrophage populations during CSS progression and assess the therapeutic implications of macrophage targeting after disease onset. In this study, we identified MHCII^+^F4/80^+^Tim4^-^ macrophages as the primary contributors to the pro-inflammatory environment in CSS, while CD206^+^F4/80^+^Tim4^+^ macrophages, with an anti-inflammatory profile, become outnumbered. Additionally, we observed an expansion of Tim4^-^ macrophages coinciding with increased hematopoietic stem progenitor cells and reduction of committed myeloid progenitors in bone marrow and spleen. Critically, macrophage targeting with clodronate liposomes at disease onset prolonged survival, while their targeting in mice with established CSS exacerbated disease severity, leading to a more dramatic loss of Tim4^+^ macrophages and an imbalance in pro- versus anti-inflammatory Tim4^−^ macrophage ratio. Our findings highlight the significance of timing in macrophage-targeted interventions for effective management of CSS and suggest potential therapeutic strategies for diseases characterized by uncontrolled inflammation, such as sepsis.

## Introduction

Cytokine storm syndrome (CSS) refers to conditions characterized by excessive immune cell activation leading to systemic inflammation, possibly occurring in patients with sepsis, autoimmune disorders, or following immune therapies. The elevated cytokine and chemokine levels released by hyperactive immune cells can induce multi organ damage, causing liver dysfunction, cardiomyopathy, kidney failure, encephalopathy, and eventually culminating with sudden death (1–4). Identifying patients early enough prior to the development of an unstoppable inflammatory storm is critical for a positive clinical outcome. Unfortunately, specific biomarkers that could predict when a patient is developing CSS are currently not available, and use of biologics might not be sufficient if organ damages have already occurred.

Macrophages are heterogeneous innate immune cells with ability to defend the body from invading pathogens, participate in tissue remodeling and wound healing, and even modulate tumor progression (5). Several studies implicated the involvement of macrophages in sepsis (6, 7) and CSS (8–11). Distinct types of macrophages have been described in these conditions, with pro-inflammatory macrophages contributing to cytokine production and T cell activation, and hemophagocytic macrophages engulfing red blood cells, white blood cells or platelets in liver, spleen and bone marrow. However, whether hemophagocytic macrophages possess pro-inflammatory or anti-inflammatory features remains to be elucidated, as evidence supporting both exist (12). Considering that pro-inflammatory and anti-inflammatory macrophage subsets, and all phenotypes in between, coexist in a variety of conditions, a detailed characterization of the macrophage profile during CSS progression is needed to design effective therapies aimed at targeting this population.

In sepsis, liver macrophages are equipped to clear pathogens. However, during disease progression, macrophages can undergo pyroptosis, a form of cell death that causes the release of excessive pro-inflammatory cytokines, thus contributing to the development of CSS and causing acute liver damage (6). The distinct functions of macrophages can be dictated by their tissue of origin (13, 14) and adaptation to external cues. In the liver, Kupffer cells (KC) are resident macrophages located in the sinusoidal lumen, involved in the clearing of foreign antigens/toxins, phagocytosing dying erythrocytes from circulation for iron recycling and maintaining immunological tolerance (15–17). Following an infection, recruitment of monocytes and their differentiation into macrophages help fighting against pathogens by producing inflammatory cytokines (18, 19). One specific marker used to recognize liver resident macrophages is Tim4, a receptor involved in efferocytosis of the apoptotic cells (20–22). The extent to which tissue-resident Tim4^+^ liver macrophages and monocyte-derived macrophages contribute to CSS progression, and whether these subsets exhibit a distinct phenotype, remains unclear.

In this study, we focused on understanding changes in liver macrophage subsets and how they correlate with disease progression. We found that Tim4^-^ macrophages with a pro-inflammatory profile dominate the liver macrophage pool during CSS progression, while the Tim4^+^ tissue resident macrophages with an anti-inflammatory profile become outnumbered. The macrophage output is accompanied by an increase in Lin^-^Sca1^+^Kit^+^ LSK hematopoietic stem cells in the bone marrow and spleen and exhaustion of committed myeloid progenitors. Strikingly, depletion of macrophages with clodronate liposomes after disease initiation leads to early mortality accompanied by drastic reduction in Tim4^+^ macrophages and higher pro- versus anti-inflammatory Tim4^-^ macrophage ratio. This study demonstrates the coexistence of different subsets of liver macrophages with Tim4^-^ contributing to disease progression, expanding during disease progression, and Tim4^+^ trying to mitigate the excessive inflammation, becoming outnumbered.

## Materials and methods

### Mice

C57/B6 *Prf^-/-^* mice were purchased from the Jackson laboratory (Bar Harbor, ME). All mice were housed *ad libitum* with free access to feed and water. Female and male animals were used for all experiments since no gender differences were noted. When comparing different groups, the same numbers of males and/or females were used in each group. The initial body weight of the mice at the start of the infection was 20 ± 2 g and mice undergoing over 20% body weight loss after infection were immediately sacrificed per animal care guidelines. All experiments were approved by Washington University School of Medicine animal care and use committee.

### LCMV-induced CSS model and clodronate treatment

2×10^5^ plaque-forming units (PFUs) of LCMV-Armstrong were administered intraperitoneally in 8-12 weeks-old *Prf^-/-^* mice. To deplete monocytes/macrophages, 100 µl of clodronate liposomes (Liposoma BV, Amsterdam, Netherlands) were injected via the tail vein at time of LCMV infection, followed by a second and a third dose every 48 hours (referred to as early clodronate treatment). A second cohort received the clodronate liposomes starting 6 days post LCMV infection followed by two additional doses given 48 hours apart (referred to as late clodronate treatment). Either phosphate buffered saline (PBS) or control liposomes (Liposoma BV, Amsterdam, Netherlands) were used as controls. Animals were monitored daily to record survival or when indicated euthanized at a specific time after LCMV infection to collect blood, bone marrow, spleen and liver samples for further analysis.

### Serum preparation

Blood was collected from anesthetized animals via heart puncture and collected into Serum-Gel Polypropylene tubes (Thermo Fisher, USA). The serum was separated after 30 min incubation at room temperature via centrifugation at 8,000 rpm for 4 min and stored at -80°C until further use.

### Bone marrow and spleen processing for cell isolation

Tibias and femurs cleaned from attached muscles were placed each in a 0.5 mL centrifuge tube with a hole punched on the bottom, and then transferred in a larger 1.7 mL centrifuge tube followed by centrifugation at 12,000 rpm for 2 min to collect the bone marrow. The harvested bone marrow was further processed to remove red blood cells using the RBC buffer (Sigma) and then either used immediately for flow cytometry or stored at -80°C in freezing medium (10% DMSO (v/v) in FBS) until further use. For spleens, a 70 µm strainer was used to filter the mashed organ to collect the single cells. The cell suspension was centrifuged at 2,500 rpm for 5 min, followed by RBC lysis and either used immediately for flow cytometry or stored at -80°C in freezing medium (10% DMSO (v/v) in FBS) until further analysis.

### Liver processing for cell isolation

Livers were collected without perfusion, chopped into fine pieces, and processed with collagenase (Roche, USA) and DNase I (Sigma, MO, USA) in α-minimum essential medium (α-MEM, Sigma, MO, USA) for 45 min at 37°C. The resulting cell suspension was filtered through a 70 µm strainer and centrifuged at 3,500 rpm for 5 min, followed RBC lysis and either used immediately for flow cytometry or stored at -80°C in freezing medium (10% DMSO (v/v) in FBS) until further analysis.

### Flow cytometry

For the analysis of the monocytes, neutrophils and macrophages, the single cell suspensions from liver, bone marrow or spleen were resuspended at a density of 5 x 10^6^ cells/mL in PBS. After blocking with anti-mouse CD16/32 blocker (Biolegend, USA) for 20 min at 4°C, the cells were stained with indicated surface markers (**Supplementary Table 1**) for 20 min at 4°C. The samples were fixed with BD cytofix buffer for 30 min at room temperature and resuspended in FACS buffer (0.5% BSA, 2mM EDTA, 0.01% NaN3 in PBS) until acquisition using the BD X-20 flow cytometer. For the analysis of hematopoietic stem and progenitor populations in the bone marrow and spleen, single cell suspensions were stained at density of 10 x 10^6^ cells for HSPC and Myeloid Progenitor Panels. Cells were incubated for 18 hours overnight at 4°C with cell surface markers for both panels with respective dilutions (**Supplementary Table 1**). Before acquisition, cells were suspended in fresh media with the viability dye 7-AAD (1:100) to exclude dead cells during the acquisition and analysis. Cell numbers were calculated based on the cell counts (using trypan blue staining) of each organ before staining for surface markers (Formula used: Population Cell number = [population % of live cells/100] * organ cell number).

### Multiplex ELISA

For analysis of cytokines and chemokines in animals receiving late clodronate treatment, LCMV-infected mice were treated every 48 hours with clodronate liposomes or PBS starting 6 days post infection and serum was collected 24 hours after the last treatment injection (11 days post infection). Cytokine and chemokine serum levels were detected using the MILLIPLEX kit (EMD MILLIPORE) as per manufacturer’s instructions and fold changes were calculated between clodronate treated and untreated controls. For analysis of fold changes in cytokines and chemokines in animals receiving early clodronate treatment versus untreated controls, we used data from samples generated in our previously published study (8), where LCMV-infected animals were treated with clodronate liposomes every 48 hours starting two days before LCMV infection, and serum was collected 8 days post infection. The rationale for analyzing the changes in cytokine levels from our previously collected samples was based on the similar survival benefits following the delivery of clodronate 2 days before or on the same day of LCMV-infection.

### Statistical analysis

Data is represented as mean +/- SEM using GraphPad Prism 10. Statistical significance was determined by unpaired two-tailed student’s *t*-test or in experiment with multiple comparisons by either one-way in conjunction with Tukey’s multiple comparison test or two-way ANOVA in conjunction with Sidak’s multiple comparison test, as indicated. For survival studies, the significance was determined by log-rank (Mantel-Cox) test. *P* value <0.05 is set as statistically significant.

## Results

### Monocyte accumulation during CSS progression correlates with expansion of multi potent progenitor subsets

**Figure 1:**
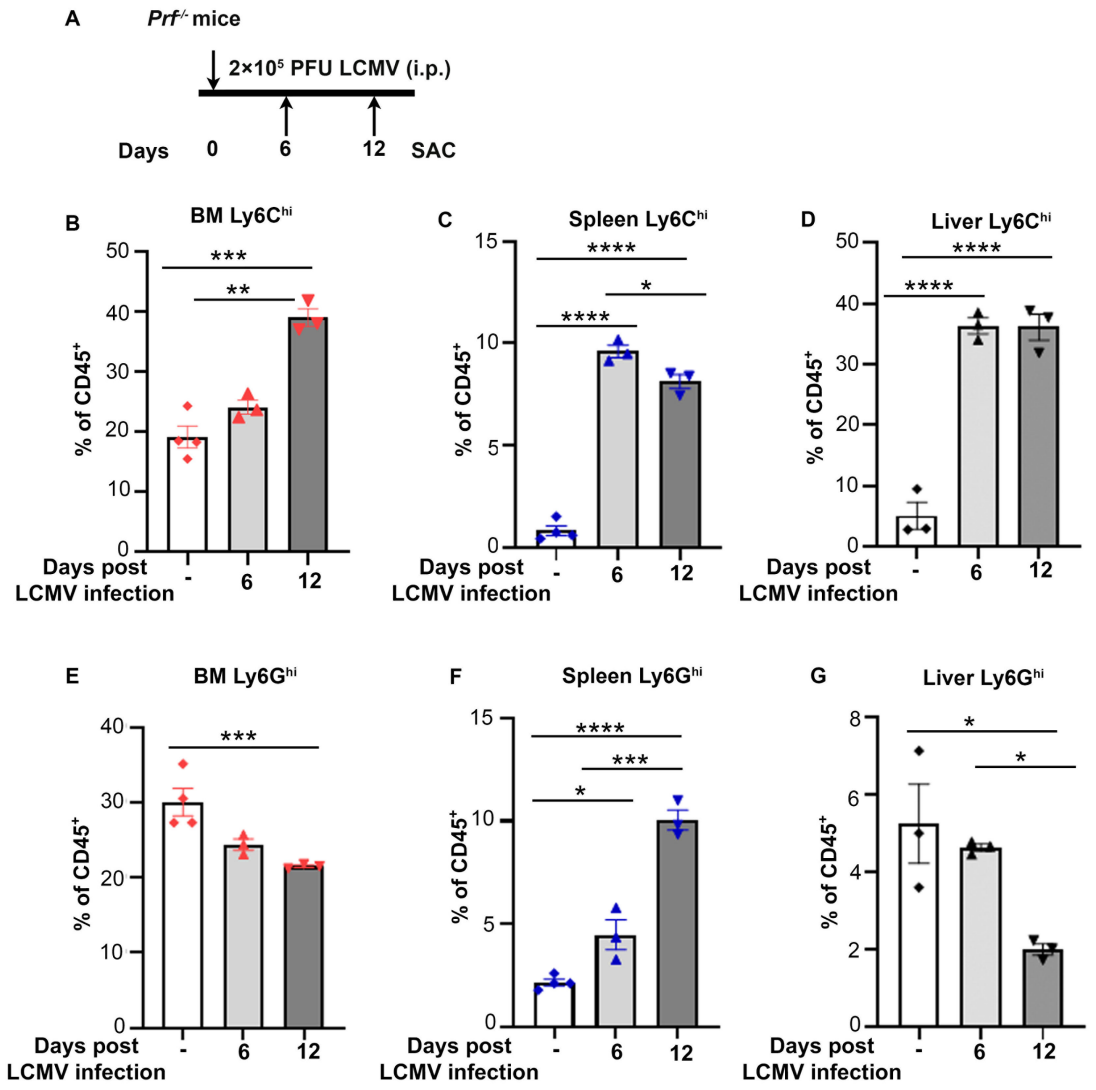
Monocytes accumulate in bone marrow, spleen and liver during CSS progression. **(A)** Schematic representation of LCMV induced CSS in *Prf^-/-^* mice. 2 x 10^5^ PFU LCMV-Armstrong was administered on day 0 and indicated organs collected on days 6 and 12 post infection for flow cytometry analysis. **(B-D)** Percentage of CD45^+^CD11b^+^Ly6G^-^Ly6C^hi^ cells out of total immune cells during CSS progression (n=3-4/group) in the bone marrow **(B)**, spleen **(C)** and liver **(D)**. **(E-G)** Percentage of CD45^+^CD11b^+^Ly6G^hi^ cells out of total immune cells during CSS progression (n=3-4/group) in the bone marrow **(E)**, spleen **(F)** and liver **(G)**. Data is presented as mean ± SEM. Statistical significance is determined by one-way ANOVA for multiple comparisons. *P < 0.05, **P < 0.01, ***P < 0.001 and ****P < 0.0001.

CSS is a complication of sepsis characterized by elevated levels of circulating inflammatory cytokines. In a mouse model of CSS consisting of LCMV infection in *Perforin^-/-^ (Prf^-/-^)* mice, which lack T cells to fight the virus, peak inflammatory responses are observed by 8 days post infection (8). To temporally characterize changes in myeloid populations during CSS progression, we collected bone marrow, spleen and liver from *Prf^-/-^* mice infected with LCMV for 6 and 12 days or non-infected controls (**Figure 1A**). Accumulation of monocytic populations (CD45^+^CD11b^+^Ly6G^-^Ly6C^hi^) was observed at all three sites (**Figures 1B–D**; **Supplementary Figures 1A–C**), with a significant increase detectable already 6 days post infection in the liver and spleen. In contrast, granulocytic populations (CD45^+^CD11b^+^Ly6G^hi^) were only increased in the spleen and decreased in bone marrow and liver (**Figures 1E–G**; **Supplementary Figures 1A–C**).

**Figure 2:**
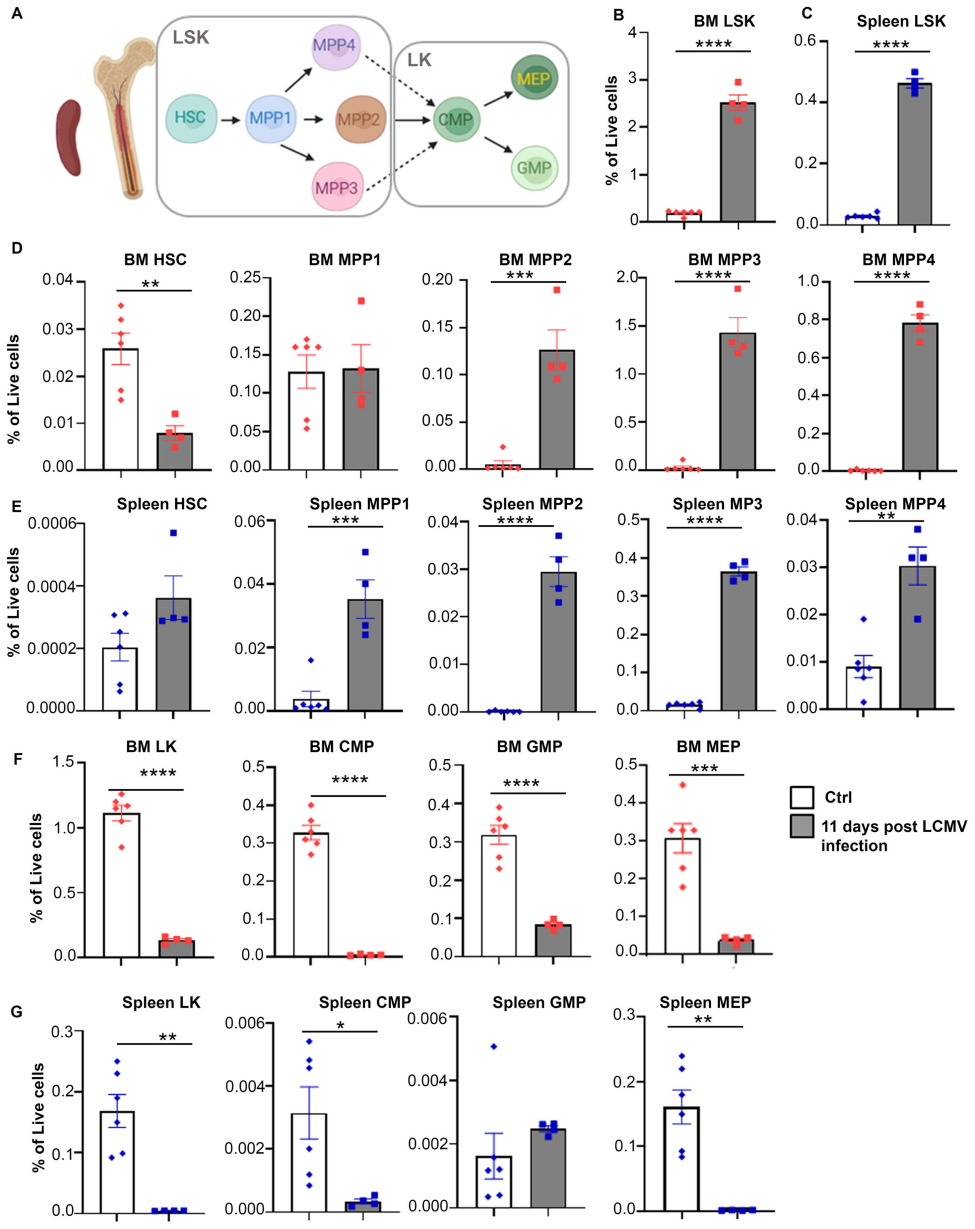
Hematopoiesis is altered with CSS progression. **(A)** Schematic illustration of hematopoietic stem cell differentiation (LSK: Lin^-^Sca1^+^cKit^+^, HSC: hematopoietic stem cell, MMP: multipotent progenitor, LK: Lin^-^Sca1^-^cKit^+^, CMP: common myeloid progenitor, MEP: megakaryocyte/erythroid progenitor, GMP: granulocyte/macrophage progenitor). **(B, C)** Percentages of LSK in bone marrow **(B)** and spleen **(C)** of *Prf^-/-^* mice on day 0 (n=6) or 11 post LCMV infection (n=4). **(D, E)** Percentage of indicated hematopoietic stem/multipotent progenitor populations out of total live cells from the bone marrow **(D)** and spleen **(E)**. **(F, G)** Percentage of indicated myeloid committed precursors out of total live cells from the bone marrow **(F)** and spleen **(G)**. Data is presented as mean ± SEM. Statistical significance is determined by two-tailed t-test. *P < 0.05, **P < 0.01, ***P < 0.001 and ****P < 0.0001.

To further investigate whether the increase in monocytic populations was driven by changes in hematopoietic stem and progenitor cells (HSPCs), which give rise to all immune cell types (**Figure 2A**), we analyzed the percentage of HSPCs and their absolute numbers in bone marrow and spleen of *Prf^-/-^* mice 11 days post LCMV infection (**Supplementary Figure 2**). Expansion of LSK (Lin^-^Sca1^+^cKit^+^) populations occurred following LCMV infection in bone marrow and spleen (**Figures 2B, C**; **Supplementary Figures 3A, B**). LSK cells were further divided into phenotypic HSCs and multipotent progenitors MPP1-4 (**Supplementary Figures 2A, B**). Strikingly, the percentage and absolute numbers of phenotypic bone marrow HSCs (Lin^-^Sca1^+^cKit^+^Flk2^-^CD150^+^CD48^-^) were drastically reduced (**Figure 2D**; **Supplementary Figure 3C**). In contrast, the percentage of splenic HSCs was unchanged (**Figure 2E**), but their absolute numbers were elevated in LCMV infected mice (**Supplementary Figure 3D**), an indication of extramedullary hematopoiesis. Additionally, we observed a significant increase in MPP2-4 percentages and absolute numbers both in bone marrow and spleen (**Figures 2D, E**; **Supplementary Figures 3C, D**). To examine changes in myeloid committed precursors, we quantified LK cells (Lin^-^Sca1^-^cKit^+^), which include Common Myeloid Precursors (CMP), Granulocyte-Monocyte Precursors (GMP), and Megakaryocyte-Erythroid Precursors (MEP) (**Figure 2A**; **Supplementary Figures 2C, D**). Interestingly, LK cells and the resulting myeloid progenitor populations (CMP, GMP, and MEP) were drastically reduced in the bone marrow and spleen following LCMV infection, an indication of exhaustion of myeloid committed progenitors (**Figures 2F, G**; **Supplementary Figures 3E, F**). These findings suggest that the expansion of mature monocytic populations occurring during LCMV infection derives from increased hematopoietic output.

### Accumulation of MHC-II^+^/Tim4^-^ macrophages and reduction of CD206^+^/Tim4^+^ macrophages occur in liver during CSS progression

**Figure 3:**
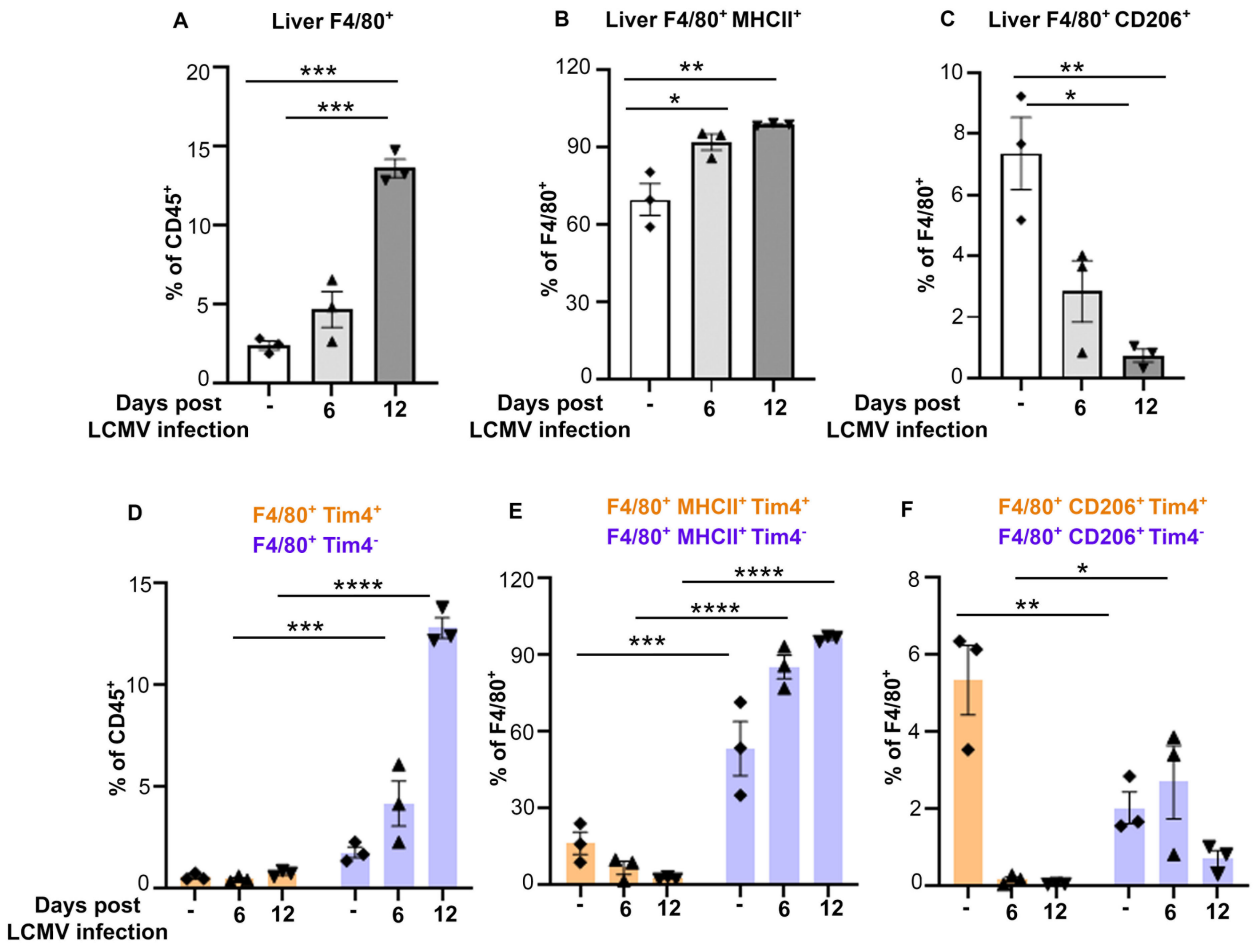
MHCII^+^/Tim4^-^ liver macrophages outnumber CD206^+^/Tim4^+^ macrophages during CSS progression. **(A-C)** Percentage of CD45^+^CD11b^+^Ly6G^-^F4/80^+^ macrophages **(A)**, expressing MHCII **(B)** or CD206 **(C)** in liver of *Prf^-/-^* mice collected on day 0, 6 or 12 post LCMV infection (n=3/group). **(D-F)** Percentages of CD45^+^CD11b^+^Ly6G^-^F4/80^+^Tim4^+/-^ populations and analysis of F4/80^+^MHCII^+^Tim4^+/-^ and F4/80^+^CD206^+^Tim4^+/-^ populations in liver of mice infected with LCMV for 0, 6 or 12 days. Tim4^+^ cells are shown in orange and Tim4^-^ in purple. Data is presented as mean ± SEM. Statistical significance is determined by one-way ANOVA **(A-C)** or two-way ANOVA **(D-F)** for multiple comparisons. *P < 0.05, **P < 0.01, ***P < 0.001 and ****P < 0.0001.

To determine whether the increase in monocytes during CSS leads to accumulation of macrophage populations, we analyzed the percentage of CD45^+^CD11b^+^Ly6G^-^F4/80^+^ macrophages in bone marrow, spleen and livers of *Prf^-/-^* mice prior to LCMV infection or 6- and 12-days thereafter. Accumulation of CD45^+^CD11b^+^ Ly6G^-^F4/80^+^ macrophages was prominent by day 12 in the liver (**Figure 3A**; **Supplementary Figure 4**), but not in bone marrow and was partially increased in the spleen (**Supplementary Figures 5A–D**).

We analyzed the percentage of classically activated/pro-inflammatory (M1-like) and alternatively activated/anti-inflammatory (M2-like) macrophages by the surface expression of MHCII and CD206 markers, respectively. We observed a significant increase in the percentage of F4/80^+^MHCII^+^ (M1-like) populations (**Figure 3B**), while F4/80^+^MHCII^-^CD206^+^ (M2-like) cells were drastically decreased during disease progression (**Figure 3C**).

To understand if the increase in F4/80^+^ populations resulted from the expansion of liver resident macrophages and/or accumulation of monocyte-derived macrophages, we analyzed by flow cytometry the percentage of Tim4^+^ macrophages, a marker mainly expressed by the tissue resident cells, compared to Tim4^-^ macrophages, which include monocyte-derived macrophages. While we did not observe major changes in F4/80^+^ Tim4^+^ macrophages (**Figure 3D**; **Supplementary Figure 4**), Tim4^-^ F4/80^+^ macrophages were twice more abundant than their Tim4^+^ counterpart in uninfected mice and that their percentage increased about 10-fold at late stages of CSS.

Further subset analysis based on MHCII and CD206 expression revealed that the MHCII^+^/M1-like macrophages were primarily derived from the Tim4^-^ macrophage population, with ~2 -fold increase from baseline by day 12 post infection. In contrast, CD206^+^/M2-like macrophages were ~2.5 fold higher in the Tim4^+^ populations compared to the Tim4^-^ counterpart at baseline, but their percentage drastically decreased following LCMV infection (**Figures 3E, F**; **Supplementary Figure 4**). These results indicate that the increase in macrophage populations in liver during CSS is due to accumulation of M1-like Tim4^-^ macrophages at the expense of M2-like Tim4^+^ macrophage population.

### Macrophage targeting by clodronate-liposome administration after CSS onset increases disease severity

**Figure 4:**
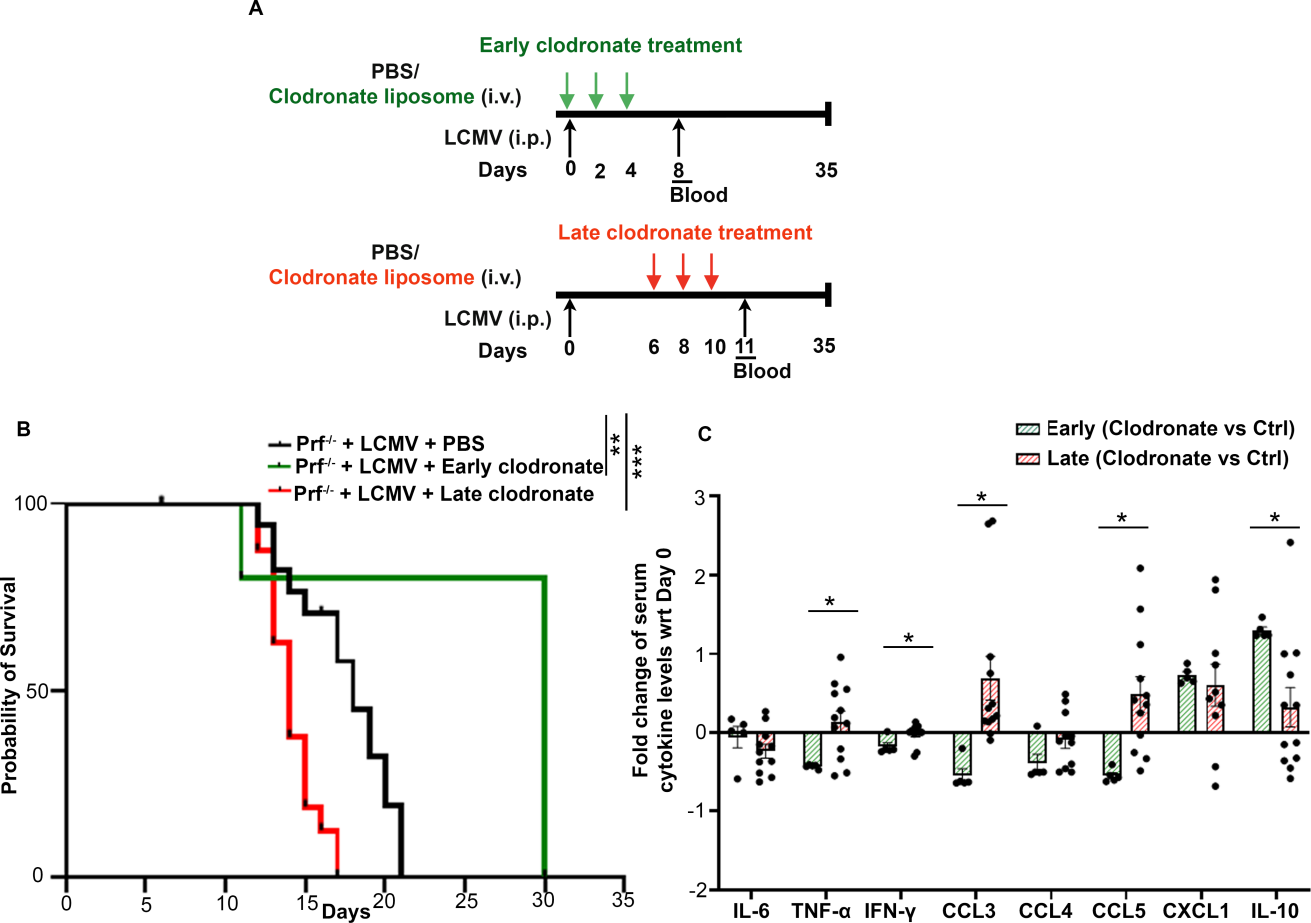
Clodronate-liposome administration after CSS onset increases disease severity. **(A)** Schematic representation of *Prf^-/-^* mice infected with LCMV and receiving clodronate-liposomes on days 0, 2 and 4 (early treatment) or on days 6, 8 and 10 (late delivery) post infection. **(B)** Probability of survival of LCMV-infected *Prf^-/-^* mice treated with PBS (n=16), receiving early (n=5) or late clodronate liposome treatment (n=16). **(C)** Fold change of indicated serum cytokines and chemokines between clodronate treated mice versus untreated controls in animals receiving early clodronate treatment from data collected in (8) or following the late clodronate delivery. Data is presented as mean ± SEM. Statistical significance was determined by Log-rank (Mantel-Cox) test **(B)** and two-tailed t-test **(C)**. *P < 0.05, **P < 0.01 and ***P < 0.001.

We previously documented that depletion of macrophages via clodronate-liposome delivery prior to CSS onset prolonged the survival of *Prf^-/-^* mice infected with LCMV (8). To better mimic the treatment of patients with established disease, we tested the therapeutic efficacy of clodronate-liposome delivery starting 6-days post-LCMV infection, followed by two doses at day 8 and 10 (referred to as late treatment) (**Figure 4A**). As controls, we used *Prf^-/-^* mice infected with LCMV receiving either PBS or clodronate-liposomes on days 0, 2 and 4 post-infection (referred to as early treatment) (**Figure 4A**). About 80% of LCMV-infected *Prf^-/-^* mice receiving early treatment survived the infection while control mice died within 21 days (**Figure 4B**), confirming our previous findings (8) in mice receiving the clodronate liposomes 2 days prior LCMV infection. Surprisingly, mice administered late clodronate treatment succumbed earlier than the untreated control group (**Figure 4B**). To better understand the causes of the early lethality, we measured the levels of circulating cytokines 24 hours after the last clodronate administration. Our previous study demonstrated that early clodronate treatment significantly decreased the circulating levels of pro-inflammatory cytokines and chemokines compared to untreated controls (8). To our surprise, late clodronate treated mice had a similar increase in pro-inflammatory cytokines IL-6, TNF-α and IFN-γ, and chemokines including CXCL1, CCL3, CCL4 and CCL5 compared to untreated animals (**Supplementary Figure 6**). IL-10, an anti-inflammatory cytokine, was also similarly elevated in both groups (**Supplementary Figure 6**). Next, we performed a direct comparative analysis and evaluated the fold change of circulating cytokines and chemokines between early clodronate-treated mice and their untreated controls from our previous study (8) versus animals receiving the late treatment and their untreated counterparts. Notably, mice receiving delayed clodronate showed significantly higher TNF-α, IFN-γ, CCL3 and CCL5 and a significant reduction in IL-10, when compared to animals receiving early clodronate treatment (**Figure 4C**) (8). This finding indicates the inefficiency of late clodronate delivery compared to early treatment and the need for an early window of intervention.

### Delayed macrophage targeting reduces Tim4^+^ macrophages and alters M1/M2 Tim4^-^ macrophage ratio

**Figure 5:**
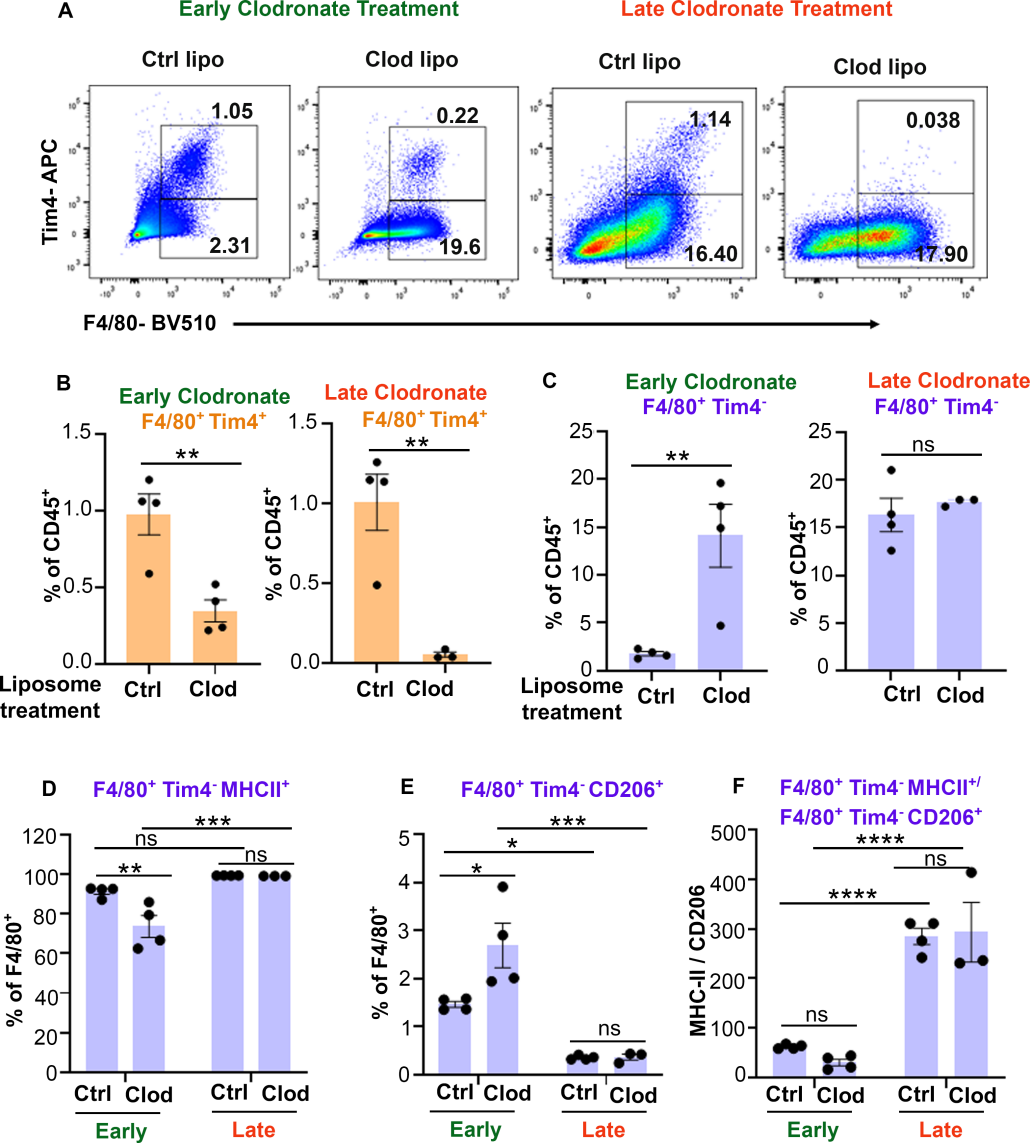
Late clodronate-liposome delivery reduces Tim4^+^ macrophages and increases the MHCII^+^/CD206^+^ macrophage ratio in liver. **(A)** Representative dot plots of CD45^+^Ly6G^-^CD11b^+^F4/80^+^Tim4^+/-^ populations in liver of LCMV-infected *Prf^-/-^* mice, sacrificed 24 hours after receiving early (0, 2 and 4) or late (6, 8 and 10) clodronate liposome treatment (n=3-4/group) and compared to control liposomes. **(B)** Percentage of F4/80^+^Tim4^+^ liver macrophage populations showing depletion efficiency following either early or late clodronate liposome treatment compared to controls. **(C-E)** Percentage of F4/80^+^Tim4^-^ macrophages **(C)**, expressing MHCII **(D)** or CD206 **(E)**, in liver of mice with CSS receiving early or late clodronate liposomes compared to control liposomes. **(F)** Ratio of MHCII^+^/CD206^+^ Tim4^-^ macrophages in liver of LCMV-infected *Prf^-/-^* mice receiving early or late clodronate liposome treatment compared to control liposomes. Data is presented as mean ± SEM. Statistical significance is determined by two-tailed t-test **(B, C)** or two-way ANOVA for multiple comparisons **(D-F)**. *P < 0.05, **P < 0.01, ***P < 0.001 and ****P < 0.0001.

To determine whether clodronate delivery at late stages of CSS progression could be targeting an anti-inflammatory macrophage subset and/or might be insufficient to suppress the exuberant expansion of pro-inflammatory macrophages, we assessed changes in Tim4^+^ and Tim4^-^ macrophage subsets along with their polarization status (**Supplementary Figures 7A, B**). We observed a significant depletion of Tim4^+^ cells with both early and late clodronate treatment (**Figures 5A, B**) confirming clodronate’s ability to target tissue resident macrophages (23, 24). Strikingly, Tim4^+^ cells were more drastically reduced in mice receiving late versus early clodronate (**Figure 5A**; **Supplementary Figure 7C**). Interestingly, not only we did not observe any depletion of Tim4^-^ macrophages in both early and late treated mice, but rather observed a significant increase in this population following early clodronate (**Figure 5C**; **Supplementary Figure 7D**). Further macrophage subset analysis revealed decreased M1-like and increased M2-like macrophages in the early clodronate treatment group versus controls. In contrast, the late clodronate treatment had nearly 100% of F4/80^+^ cells expressing M1-like markers, similarly to untreated controls treatment (**Figures 5D, E**). Analysis of M1/M2 macrophage ratio confirmed the striking bias towards an M1 pro-inflammatory macrophage phenotype in animals receiving late clodronate treatment (**Figure 5F**).

## Discussion

In this study, by using a well-established virus-induced CSS mouse model, we performed a temporal and phenotypic characterization of the macrophage subsets during disease progression. We focused on liver macrophages due to their ability in causing liver damage in CSS and sepsis. We demonstrated that the MHCII^+^F4/80^+^Tim4^-^ macrophages represent the most abundant subset in liver contributing to the pro-inflammatory milieu during CSS progression, while CD206^+^F4/80^+^Tim4^+^ macrophages with an anti-inflammatory phenotype are drastically outnumbered. The expansion of the Tim4^-^ macrophage pool is accompanied by increased LSK hematopoietic stem cells and exhaustion of committed myeloid progenitors. Importantly, we further show that in contrast to clodronate liposome delivery at disease onset, clodronate treatment in advanced stages of CSS progression accelerates mortality due to an increase in the M1/M2 macrophage ratio.

The radically distinct effects of targeting macrophages with clodronate liposomes during the initial phases of CSS development versus more advanced stages, raises the question as to why clodronate delivery after disease onset has no therapeutic benefits. Several animal studies focusing on targeting monocytes/macrophages or macrophage-secreted cytokines have shown successful protection from CSS development (25–27). Clodronate liposome delivery starting prior to the induction of sepsis has also shown reduction in pro-inflammatory cytokine production (28, 29). However, treatment is often delayed in patients. Surprisingly, and contrary to our expectations, mice receiving clodronate liposomes 6 days after LCMV infection, when monocyte expansion and macrophage accumulation have already occurred, not only do not show any therapeutic benefits but rather exhibit earlier mortality compared to untreated mice. The compromised therapeutic efficacy of delayed clodronate delivery can be attributed to the inability of this approach to halt the accumulation of Tim4^-^MHCII^+^ macrophages, likely due to an excessive influx of Ly6C^hi^ monocytes into the liver, and/or to the preferential targeting of CD206^+^Tim4^+^ anti-inflammatory macrophages. First, we noticed that the percentage of Tim4^-^MHCII^+^ liver macrophages is significantly lower in the early clodronate versus the late treatment groups. We also noted a significant increase in Tim4^-^CD206^+^ liver macrophages following early clodronate treatment, with ~60% of F4/80^+^Tim4^-^ macrophages expressing MHCII and only 2-3% expressing the anti-inflammatory marker CD206. Yet, these animals show prolonged survival to LCMV, increased IL-10 in circulation and reduced pro-inflammatory cytokines compared to untreated mice (8). These findings suggest that the small pool of M2-like macrophages might have more potent anti-inflammatory effects than the larger pool of M1-like macrophages. Similarly, in rheumatoid arthritis patients, accumulation of pro-inflammatory M1 macrophages contribute to an imbalanced M1/M2 ratio during the disease flare, however in the remission phase, CD206^+^ M2 macrophages positively contribute to the disease resolution via releasing anti-inflammatory cytokines (30). Interestingly, mice receiving delayed clodronate liposomes, have more than 90% of F4/80^+^ Tim4^-^ macrophages expressing M1-like markers and only 0.5% macrophages expressing M2-like markers. The drastic reduction in Tim4^+^ macrophages and the increased M1/M2 Tim4^-^ ratio raises the possibility that the delayed treatment inefficiency might be due to lack of a strong anti-inflammatory response. Indeed, IL-10 levels are only increased in the early clodronate treatment group, but not in the late treatment mice. Notably, deficiency of IL-10 was shown to significantly worsen CpG-induced CSS to a lethal phenotype and lead the onset of an irreversible septic shock in a mouse model of sepsis induced by cecal ligation and puncture (31, 32). These results suggests that rather than finding therapeutic options aimed at limiting monocytes and macrophage recruitment to tissues, emphasis could be placed to therapies that can polarize even a small proportion of M1 macrophages into M2-like cells to produce higher levels of IL-10 and other anti-inflammatory cytokines.

Tissue of origin, in addition to external factors, dictates the macrophage phenotype. We used Tim4 expression to distinguish liver resident cells from monocyte-derived macrophages (33). Tissue resident macrophages have been shown to express an anti-inflammatory signature and genes related to tissue repair in various diseases. Strikingly, in our CSS model the tissue resident Tim4^+^ cells are drastically decreased. Similar findings were reported in non-alcoholic steatohepatitis (NASH), where the mature TIM4^pos^ Kupffer cells decreased in number, while monocyte derived Tim4^neg^ macrophages accumulated (33). Nevertheless, the bone-marrow derived macrophages acquired expression of Kupffer cell like markers and contributed to increase liver fibrosis. In our model, all Tim4^+^ cells are drastically reduced following LCMV infection, while their Tim4^-^ counterpart expressing both M1-like and M2-like markers are proportionally increased. Thus, we cannot exclude that a proportion of Tim4^-^ cells acquires tissue repair properties, an assumption that is supported by the increase in M2-like cells and survival benefits in the early clodronate treatment cohort. This finding supports the notion that monocyte-derived macrophages can be reprogrammed to mitigate the excessive inflammation in CSS.

The drivers of full-blown CSS in patients, especially those with auto-inflammatory conditions, are not completely known. Infections have been considered as plausible causes for CSS initiation (34). Elevated IFN-γ levels have been shown to induce the proliferation of bone marrow HSCs following bacterial infection and drive a bias toward myeloid cell expansion (35). According to the striking increase in IFN-γ levels in our LCMV-induced CSS model, we also found expansion of LSK stem cell populations. Subset analysis, however, revealed a decrease in phenotypic HSCs but accumulation of the multipotent progenitors. Strikingly, the committed myeloid progenitors CMP and GMP, were drastically reduced, a likely consequence of the elevated demand for monocyte/macrophage expansion. Accumulation of monocytes in the liver and spleen has also been observed in the CpG-induced CSS model, however rather than increases in bone marrow monocyte progenitors, the TLR9-dependent model was rather dependent on the expansion of extramedullary myeloid progenitors (36). Our findings are more in line with changes in bone marrow hematopoiesis following chronic infections, as shown by the higher frequencies and numbers of LSK stem cell populations but depletion of phenotypic HSCs and exhaustion of committed progenitors (37). During severe inflammatory responses like polymicrobial sepsis, the myeloid rebound is attributed to enhanced output from the MPP proliferation and differentiation rather than HSCs (38). Therefore, it would be important to understand if patients who suffer from multiple CSS episodes and/or show predisposition to CSS might have long lasting hematopoietic abnormalities that drive excessive myelopoiesis following submissive infection or even in the absence of known causes.

This study has few limitations that warrant consideration. First, we only used the LCMV-induced CSS model, chosen because of its severity. Our previous work using both the LCMV-induced CSS and the CpG-induced CSS confirmed expansion of spleen and liver macrophages in both settings (8). Furthermore, loss of Tmem178, a negative regulator of M1 macrophage polarization, increased disease severity in both models (39). Our macrophage profiling is also in line with other injury and inflammatory models, including LPS-induced CSS, thus we anticipate that considerations about the timing of macrophage-directed treatment initiations should be applicable to other macrophage-driven inflammatory conditions.

Second, we used clodronate liposome delivery to target macrophages. This approach is known to preferentially deplete tissue resident macrophages and has been successfully used to protect mice from various macrophage-dependent inflammatory conditions. We previously compared the protective effects of clodronate liposome delivery and anti-CSF1 neutralizing antibody, that depletes circulating monocytes. Interestingly, both approaches when initiated at disease onset, prolonged the survival of *Prf^-/-^* mice to LCMV infection and reduced inflammatory cytokines levels. However, the clodronate liposome treatment had a more profound impact on reducing circulating chemokines and increasing IL-10 compared to anti-CSF-1. This observation prompted us to use clodronate liposomes in this study. Future work will need to determine whether CSF-1 blockade or other macrophage targeting modalities, initiated after CSS onset, worsen disease severity similarly to the late clodronate liposome delivery.

Finally, the use of scRNAseq approaches and/or various lineage tracing mouse models to identify changes in macrophage subsets have not been utilized in our study. Nevertheless, the use of common M1 and M2 markers along with markers expressed by tissue resident and monocyte-derived macrophage populations, allowed us to conclude that Tim4^-^ macrophages are the dominant subset expanding during CSS, mostly expressing MHCII but also gradually acquiring CD206 expression, and thus possibly attempting to contribute to disease resolution especially upon early clodronate treatment. Although we cannot completely exclude biases in the interpretation of our results, the use of multiple approaches including survival curves, macrophage profiling, and changes in cytokine levels demonstrate the therapeutic inefficacy of delayed clodronate delivery when compared to early treatment.

In conclusion, our study highlights the significance of timing in macrophage-targeted interventions for effective management of CSS. Furthermore, enhancing anti-inflammatory macrophage subsets may offer superior clinical outcomes for severe inflammatory diseases, including sepsis, compared to targeting pro-inflammatory macrophages. These insights have the potential to transform therapeutic strategies for conditions marked by uncontrolled inflammation.

## Data Availability

The raw data supporting the conclusions of this article will be made available by the authors, without undue reservation.
