# Supplementary material for: Delayed macrophage targeting by clodronate liposomes worsens the progression of cytokine storm syndrome
Source: Front Immunol. 2024 Oct 28;15:1477449. doi: 10.3389/fimmu.2024.1477449 (PMC11550973; doi:10.3389/fimmu.2024.1477449)
Supplement: Supplementary file 1 [file DataSheet1.pdf]

Supplementary Figures and Legends

Supplementary Fig 1

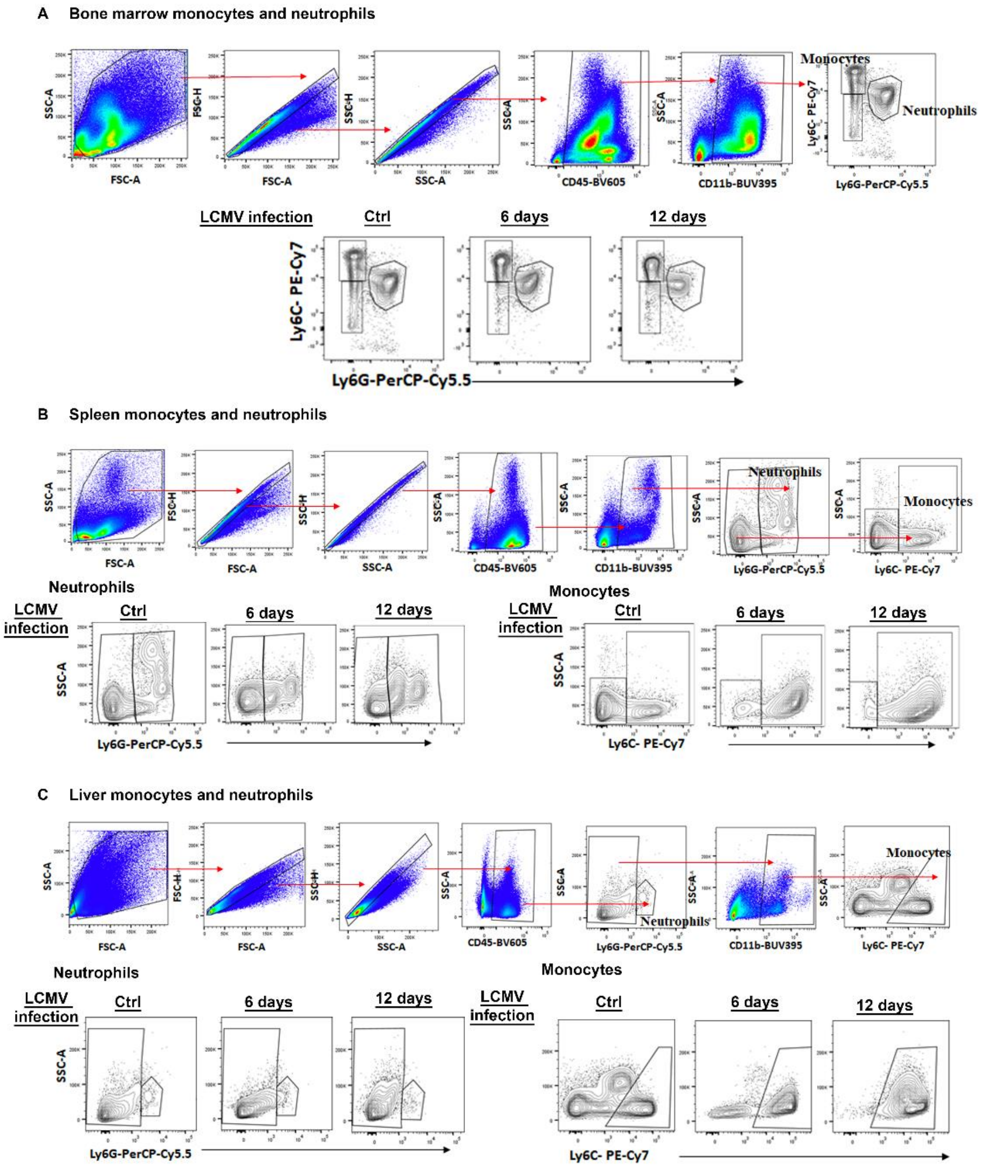

Sup figure 1. (A-C) Gating strategy and representative images for flow analysis of CD45$^+$CD11b$^+$Ly6G$^-$Ly6C^{hi} monocytes and CD45$^+$CD11b$^+$Ly6G^{hi} neutrophils in bone marrow (A), spleen (B) and liver (C) of *Prf*$^{-/-}$ mice infected for 6 and 12 days with LCMV. Mice with no LCMV infection were used as controls.

Supplementary Fig 2

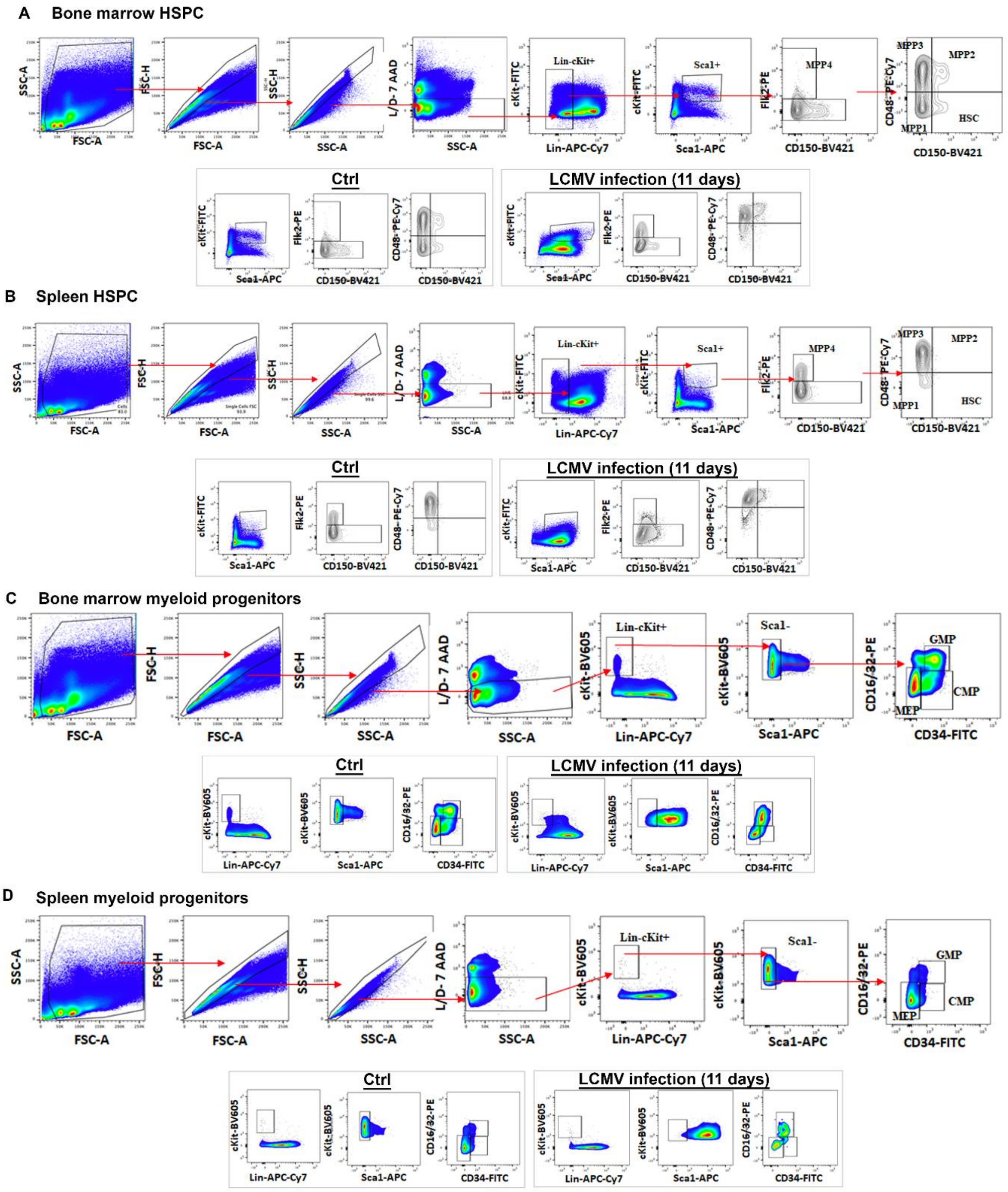

Sup figure 2. (A-D) Gating strategy and representative images of flow analysis of hematopoietic stem and progenitor cells (HSPCs) (A, B) and myeloid committed precursors (C, D) in the bone marrow and spleen from *Prf*$^{-/-}$ mice on day 0 (n=6) or 11 days post LCMV infection (n=4).

Supplementary Fig 3

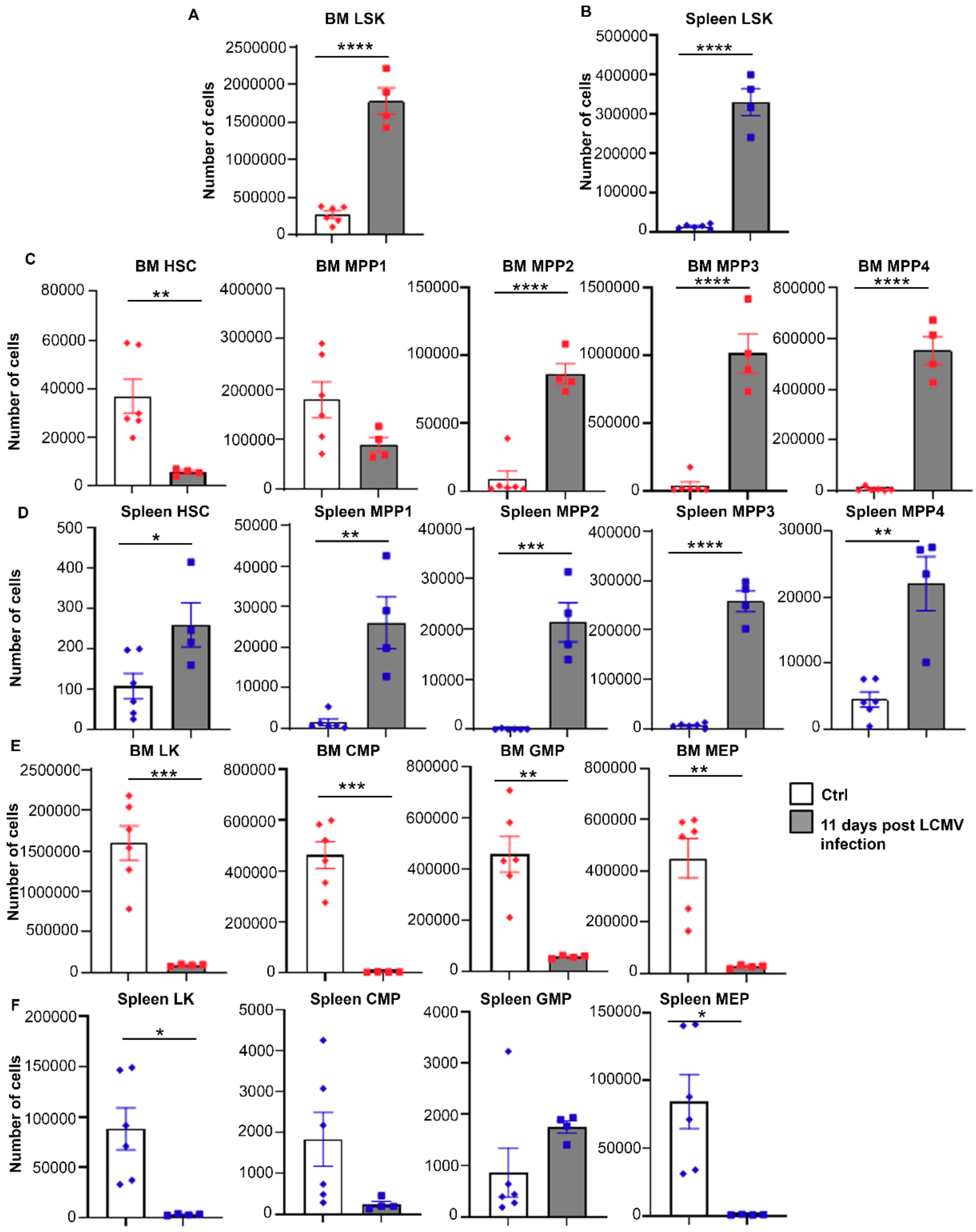

Sup figure 3. Changes in number of hematopoietic stem and progenitor cells (HSPCs) in the bone marrow (BM) and spleen during CSS progression. **(A-B)** Bone marrow (A) and spleen (B) cells collected from *Prf*$^{-/-}$ mice on day 0 (n=6) or 11 days post LCMV infection (n=4) and subjected to

flow cytometry to assess the numbers of $Lin^{-}Sca1^{+}cKit^{+}$ populations out of live cells. **(C-D)** Numbers of indicated hematopoietic stem/multipotent progenitor populations out of total live cells from the bone marrow (C) and spleen (D). **(E-F)** Numbers of indicated myeloid committed precursors out of total live cells from the bone marrow (E) and spleen (F). Data is presented as mean ± SEM. Statistical significance is determined by two-tailed t-test. *$P < 0.05$, **$P < 0.01$, ***$P < 0.001$ and ****$P < 0.0001$.

Supplementary Fig 4

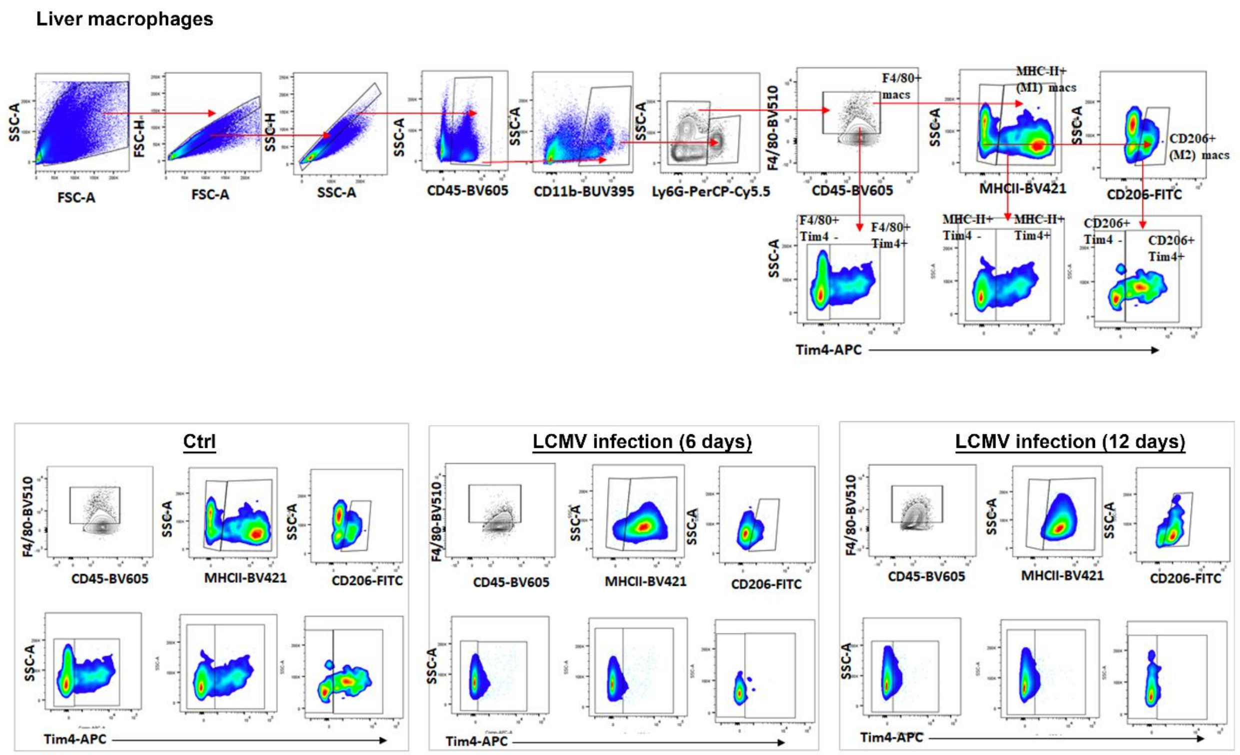

Sup figure 4. Gating strategy and representative images of flow analysis of CD45$^+$CD11b$^+$Ly6G$^-$F4/80$^+$, CD45$^+$CD11b$^+$Ly6G$^-$F4/80$^+$MHCII$^+$ and CD45$^+$CD11b$^+$Ly6G$^-$F4/80$^+$MHCII$^-$CD206$^+$ macrophage populations in liver samples collected from *Prf*$^{-/-}$ mice on day 0, 6 and 12 days post LCMV infection (n=3/group).

Supplementary Fig 5

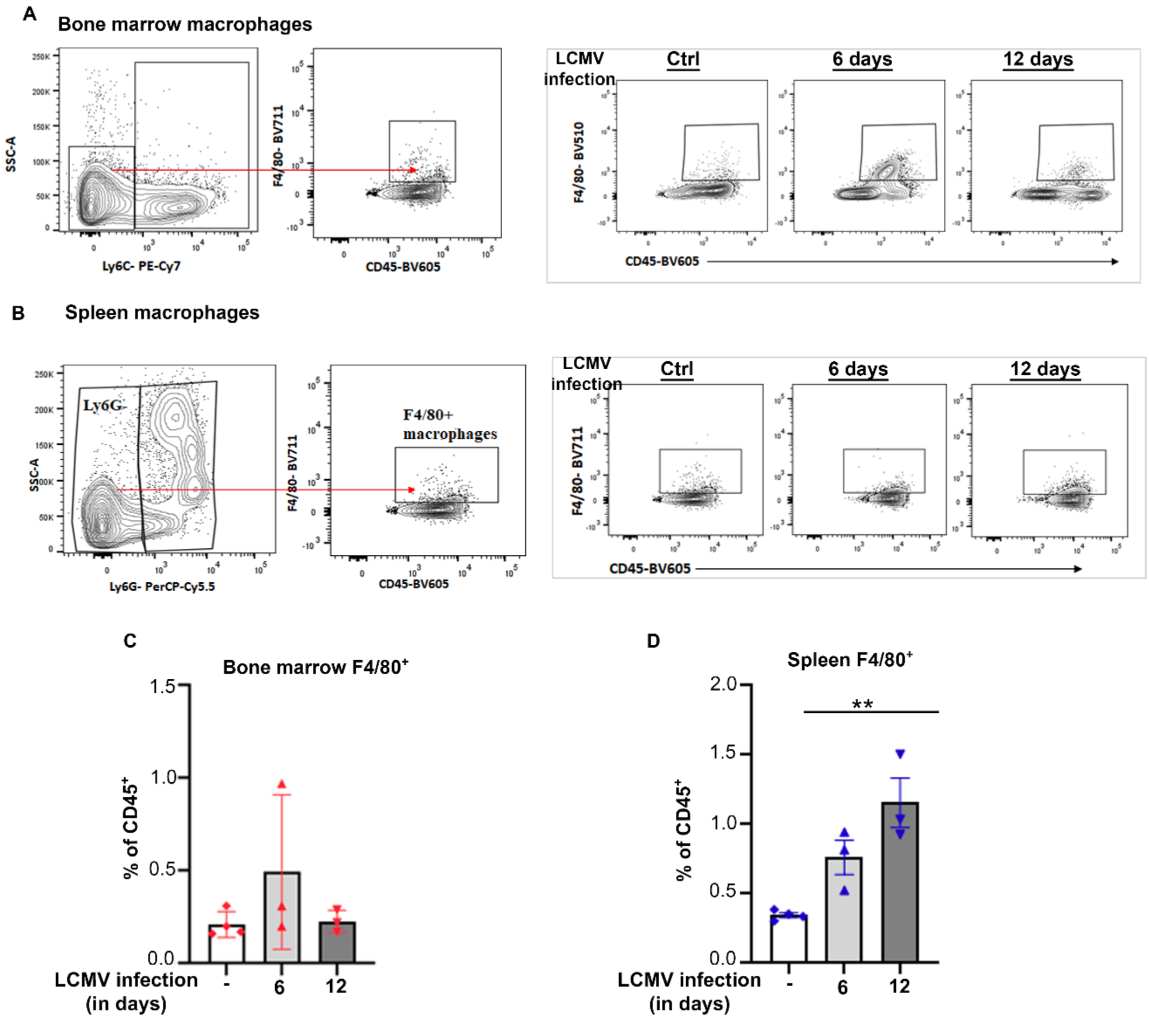

Sup figure 5. Flow cytometric analysis of macrophage populations in bone marrow and spleen of *Prf*$^{-/-}$ mice on day 0, 6 and 12 days after LCMV infection (n=3-4/group). **(A-B)** Gating strategy and representative images of flow analysis of $CD45^+CD11b^+Ly6G^-Ly6C^-F4/80^+$ macrophage populations in bone marrow (A) and spleen (B). **(C-D)** Percentage of $CD45^+CD11b^+Ly6G^-Ly6C^-F4/80^+$ cells out of total immune cells in the bone marrow (C) and spleen (D). Data is presented as mean ± SEM. Statistical significance is determined by one-way ANOVA for multiple comparisons. $**P < 0.01$.

Supplementary Fig 6

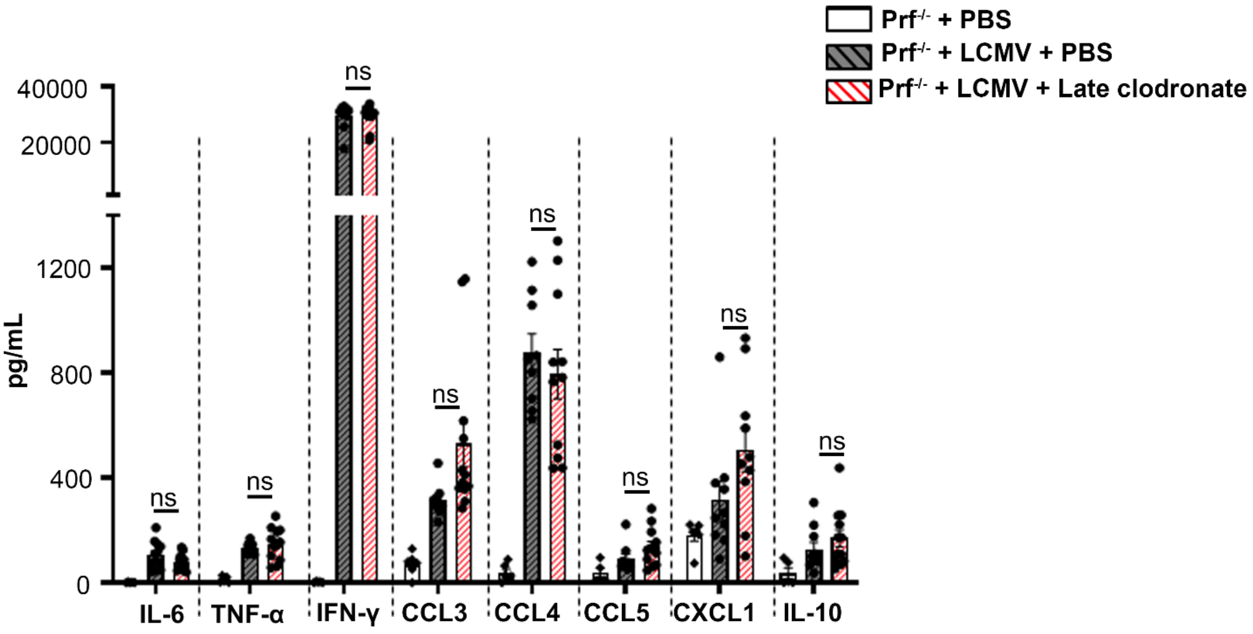

Sup figure 6. Serum levels of indicated cytokines and chemokines analyzed 5 days after late clodronate treatment initiation by Milliplex assay. Data is presented as mean ± SEM. Statistical significance was determined by two-way ANOVA.

Supplementary Fig 7

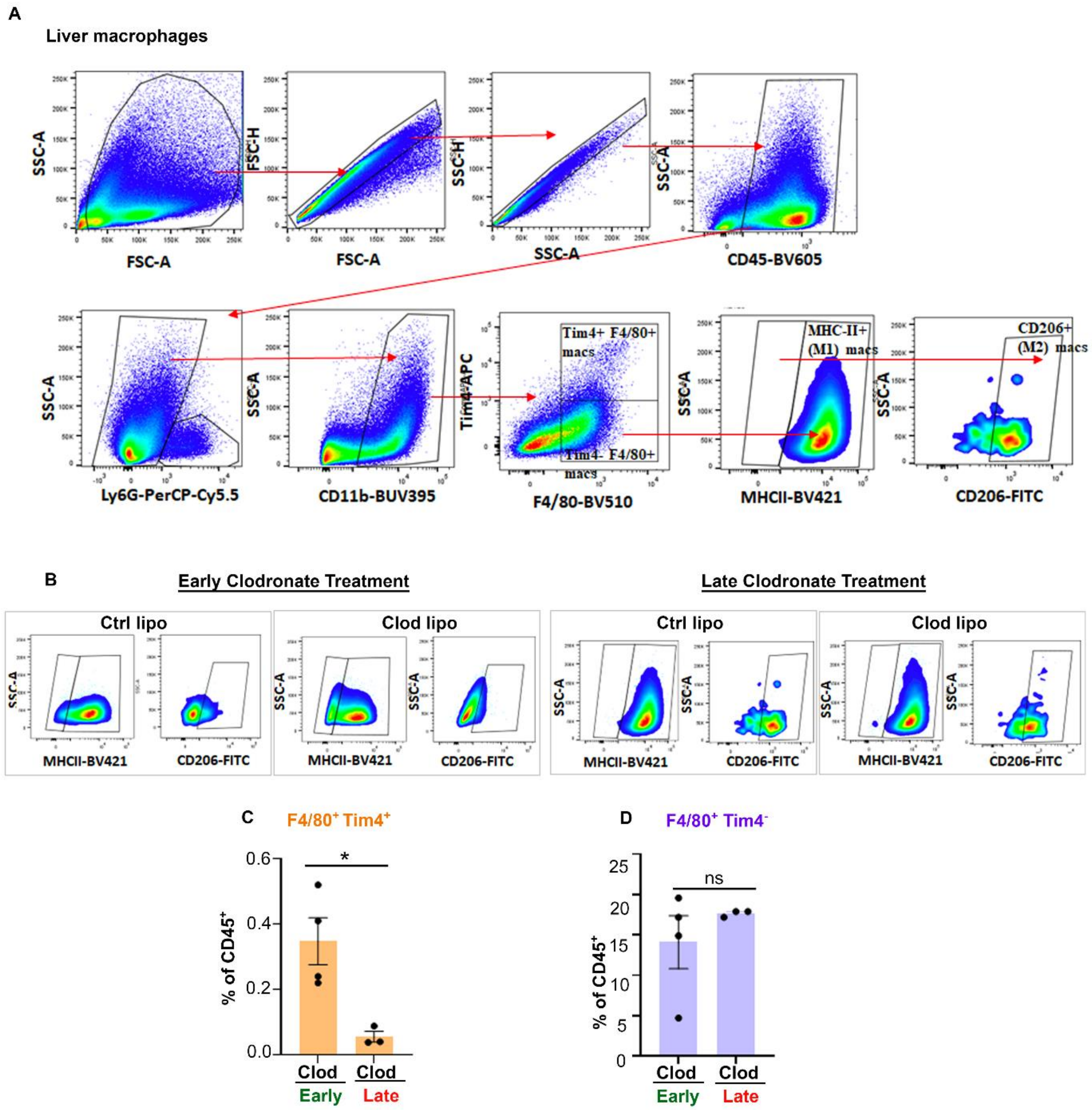

Sup figure 7. (A-B) Gating strategy and representative images of flow analysis of CD45$^+$Ly6G$^-$CD11b$^+$F4/80$^+$Tim4$^{+/-}$, CD45$^+$Ly6G$^-$CD11b$^+$F4/80$^+$Tim4$^-$MHCII$^+$ and CD45$^+$Ly6G$^-$CD11b$^+$F4/80$^+$Tim4$^-$MHCII$^-$CD206$^+$ macrophage populations in liver samples collected from *Prf*$^{-/-}$ mice treated with either control liposome or clodronate liposome at day 0, 2, 4 (early treatment) post-LCMV infection (n=4/group) and treated with control liposome or clodronate liposomes on

day 6, 8, 10 (late treatment) post-LCMV infection (n=3-4/group). **(C-D)** Percentage of $F4/80^{+}Tim4^{+}$ (C) and $F4/80^{+}Tim4^{-}$ (D) liver macrophage populations following either early or late clodronate liposome treatment (n=3-4/group). Data is presented as mean ± SEM. Statistical significance is determined by two-tailed t-test (C, D). $*P < 0.05$.

Supplemental material and methods

Supplementary Table 1

Antibody	Fluorochrome	Clone	Company	Dilution
CD45	BV605	30-F11	Biolegend	1:400
CD11b	BUV395	M1/70	BD Biosciences	1:400
Ly6G	PerCP-Cy5.5	1A8	Biolegend	1:200
Ly6C	PE-Cy7	HK1.4	Biolegend	1:200
F4/80	BV510	BM8	Biolegend	1:200
MHCII	BV421	M5/114.15.2	BD Biosciences	1:200
CD206	FITC	MR5D3	Bio-Rad	1:100
Tim4	Alexafluor 647	R4-54/RMT4-54	Biolegend	1:50
Ly6G (Gr1)	APC Cy7	RB6-8C5	Biolegend	1:100
Ter119	APC Cy7	TER-119	Biolegend	1:100
CD11b	APC Cy7	M1/70	Biolegend	1:100
CD3e	APC Cy7	145-2C11	BD Biosciences	1:100
B220	APC Cy7	RA2-6B2	Biolegend	1:100
Sca1 (Ly-6A/E)	APC	E13-161.7	Biolegend	1:100
cKit (CD117)	FITC	2B8	Biolegend	1:100
cKit (CD117)	BV605	2B8	Biolegend	1:100
CD135	PE	A2F10.1	Biolegend	1:50
CD150	BV421	TC15-12F12.2	Biolegend	1:100
CD48	PE Cy7	HM48-1	Invitrogen	1:100
CD16/32	PE	93	Biolegend	1:50
CD34	FITC	RAM34	Invitrogen	1:50

Sup Table 1 List of flow antibodies used, their clone, dilution used and manufacturer’s details.
